# Book Reviews

**Published:** 1831-02

**Authors:** 


### CRITICAL ANALYSES.

Quæ laudanda forent, et quæ culpanda, vicissim
Illa, prius, cretâ; mox hæc, carbone, *notamus.*—Persius.

*The Dublin Hospital Reports and Communications in Medicine and Surgery.* Vol. V.—8vo. pp. 640. Hodges and Smith; Dublin, 1830.

In this volume there are various papers which claim our attention, both on account of their pathological and practical importance.

The first communication is a Clinical Report of Cases in the Medical Wards of the Meath Hospital, in 1828 and 1829, by Drs. Graves and Stokes. Some interesting examples of diseases of the arterial system are recorded. The first case is rather unusual, and, as it is briefly detailed, we give it entire.

"*Gangrene and Paralysis of the right lower Extremity, arising from Disease of the Femoral and Iliac Arteries.*

"Patrick Magrath, aged forty-four, of a strong habit, was admitted on the 7th of February, 1829, labouring under loss of power of the right lower extremity.

"For the last six months this man had been exposed to severe hardships. In the beginning of December 1828, he was first affected with alternating sensations of cold and burning heat in the toes of the right foot. The same sensations, soon after, were felt in the leg, accompanied with formications, and diminished power of the limb. Pains in the foot next supervened, and in the course of a month the part became cold, and was totally deprived of sensation.

"On the day of his admission, he had attempted to walk to the hospital, when the pain suddenly extended to the calf of the leg with violence. From this time he lost all power of motion in the leg.

"The constitutional symptoms since the beginning of this disease were prostration of strength, anorexia, and constant thirst.

"On admission, his intellects were perfect, and the temperature of the body, with the exception of the affected limb, natural. The pain had extended to the thigh during the night: pulse ninety-six, small and soft. On examining the limb, we found its temperature to be about 58° of Fahrenheit, and observed some œdema at the ankle and foot. There was complete loss of sensation from the middle of the thigh to the toes; the patient could rotate the thigh slightly, but no other voluntary motion of the limb was possible. The femoral artery appeared like a hard cord; it was painful on pressure, and no pulsation could be felt in the vessel. We further discovered, by the assistance of the stethoscope, that pulsation was wanting in the external and common iliac arteries on this side, while that of the left iliacs was plainly perceptible.

"From these observations we came to the conclusion, that the right common and external iliacs, and the femoral artery, were in a state of permanent obstruction, which would account for the state of the limb.

"Warmth was applied to the limb, and opiates exhibited. In the course of the night the limb became of the natural temperature, the œdematous swelling extended to the hip, purplish patches adpeared at the ham, and the thigh became painful on pressure. Leeches were applied in abundance, and opium freely administered. On the 10th the thigh was more swollen, and presented many vesications; considerable tenderness; temperature 88°. He died the following morning.

"*Dissection.* No emaciation; the right lower extremity swollen and of a purple colour.

"The brain, lungs, and abdominal viscera, were carefully examined, but nothing remarkable was observed, except that all these parts were unusually exsanguineous. A few crude tubercles were found at the superior portions of both lungs.

"The heart presented the left ventricle in the state of active aneurism, with some thickening and opacity of the aortic valves. The ascending portion and arch of the aorta were perfectly healthy, nor could any disease be discovered in the carotid or subclavian arteries, but in the innominata we observed some red patches where the lining membrane was thickened and softened. The descending aorta was healthy to within six inches from its bifurcation: here a slender red fibrinous clot was found stretching nearly to the bifurcation; beneath this clot the lining membrane was of a deep red colour, thickened, and soft.

"The right common iliac, when viewed externally, appeared distended and livid; the left apparently healthy. On slitting down to the bifurcation, we found that the former vessel was completely plugged up from its origin by a dark clot, which extended to the external and internal iliacs, and also engaged the gluteal and obturator arteries. The same disease was found in the femoral and profunda, and extended to the origin of the anterior and posterior tibial arteries, which vessels, including the peroneal, presented a similar appearance as far as they could be traced.

"Along the course of the diseased vessels, the lining membrane of the artery was found soft and thickened. It had a somewhat villous appearance, and greatly resembled an inflamed mucous membrane. In some portions the clot was separated from the vessel by a layer of dark coloured puriform matter, in others it was adherent. The clot in the tibial arteries was not red, and much firmer than in the femoral and iliac arteries.

"In the left common iliac we found the lining membrane of a deep red colour, and the vessel contained some portions of coagulable lymph. The external iliac, and femoral arteries of this side were perfectly healthy.

"No disease whatever could be detected in the veins of the affected limb.

"A large portion of the vasti and rectus muscles was hardened, and deprived of colouring matter. The cellular tissue œdematous: periosteum red, but not softened." (P. 1.)

It is presumed, that in this case the disease had first commenced in the extreme branches of the arteries of the foot, and had gradually extended from below upwards. The symptoms favor this opinion: the numbness and alternating sensations of heat and cold, occurring first in the toes, and afterwards engaging the foot and leg; the extension of the pain upwards, and the coldness of the foot, existing at a time when the thigh preserved its natural temperature, all point out that the obstruction did not, in the first instance, commence in the larger trunks. But it is remarked, that the appearances on dissection furnish a still more satisfactory evidence of the truth of this opinion. The consistence of the clot decreased from below upwards. In the lower portions of the arteries of the leg, it was extremely firm and pale, while in the femoral and iliac arteries it was soft and red. Here also the lining membrane of the vessels showed marks of recent disease, not observable in the arteries of the leg, namely, redness, softening, and puriform exudation. The existence of redness and softening, with the presence of a coagulum in the lower portion of the aorta, and in the left common iliac, make it highly probable that here was the latest effect of the disease; and that, had the patient lived longer, the aorta itself would have become obstructed.

"The occurrence of coloured clots adhering to and derived from the inflamed portions of the aorta and left common iliac, in which the current of the blood had flowed freely, is worthy of notice, and seems to suggest the idea, that the fibrinous coagulum found in inflamed arteries, differs from that of aneurismal sacs, and is not altogether derived from the coagulation of blood arrested in its course in consequence of the obstruction. Indeed, it is highly probable that the exudation of lymph from the inflamed internal tunic of the artery, is the first cause of obstruction to the flow of blood through the diseased vessel. In the smaller trunks it must very soon fill up the caliber of the artery; in the larger, the continued clot probably results from a double source, exuded lymph and coagulated blood.

"In this case the extreme coldness of the limb pointed out, in the first instance, that the circulation was obstructed. Coldness occurs in some cases of paralysis from disease of the nervous system, but it is slight; here the temperature of the affected limb was 30° below the natural standard. This great coldness, and the slight and but little extended œdema observable on the admission of the patient, showed that the obstruction existed in the arterial rather than in the venous system, and this was borne out by the absence of pulsation in the femoral artery, as observed by the touch, and in the iliacs by auscultation. The latter observation, which we believe to be the first of the kind, was made with great ease and certainty. Having traced the pulsation of the aorta with the stethoscope, we followed the course of the vessel to the umbilical region, where the pulsation could be distinctly heard passing to the left side in the direction of the common iliac. This, however, was altogether wanting in the course of the right common iliac; and as no tumor existed in this situation, it was plain that the absence of pulsation denoted obstruction of the right common iliac artery.

"One of the most interesting circumstances in the case, is the occurrence of inflammatory action in the cellular tissue and skin of the affected limb. It is evident, that as long as the current of blood into the limb took place through the natural channels, the circulation was diminished in proportion as the arterial ramifications became diseased; the extreme parts being first affected, and afterwards the whole limb. During this state of things, the want of feeling and coldness kept pace with the progress of the arteritis.

"But when the inflammation had caused a total obliteration of all the arterial trunks leading from the common iliac of the affected side, nature appears to have made an effort, by means of anastomosing branches derived from the healthy arteries of the opposite side, to restore the circulation in the limb, in the same way as happens in cases where the common iliac has been tied. That this effort was successful may be inferred from the restoration of warmth and sensibility to the limb; and it is worthy of observation, that in this and similar cases, where a collateral circulation has been just established, the danger to be apprehended appears to arise, as in frost-bitten parts too suddenly restored, not from a deficient, but an over-active circulation in the affected extremity, which manifested evident symptoms of inflammation, such as heat, pain, tenderness, œdema, vesications, and superficial gangrene. The latter we consider in our case to have been evidently the consequence of inflammation, and it is probable that the gangrene observed in other cases of arteritis arises from the same cause, and not, as Andral seems to think, from a deficient circulation, as he compares it to gangrena senilis, from ossification of the arteries.

"If this view of the subject be correct, it evidently points out the impropriety of continuing the application of warmth after we observe that the collateral circulation has commenced, for the same reason that such applications are improper or even dangerous in the case of frost-bitten parts. Gentle warmth is in such cases at first advantageous; but when the restoration of the circulation has commenced, our efforts should be to moderate, not increase it; a suggestion that may prove useful after the operation for aneurism.

"In the advanced stages of this disease, the diagnosis is not difficult; there is paralysis, but this has not been preceded by symptoms of cerebral or spinal disease, and the intellects remain undisturbed. To this, the feeble pulsation, or its complete absence in the arteries of the limb, are to be added, and no difficulty will be experienced in recognising the disease.

"In its early stages the diagnosis is more difficult. Here, however, an accurate comparison of the temperature of both limbs, and the force of the arterial pulsations, may perhaps lead to a discovery of the disease soon after its commencement, and thus enable us to arrest the progress of the inflammation. At all events, the disease might be checked, if not cured, so as to allow the anastomosing vessels time to take on the supplementary action." (P. 14.)

For somewhat similar cases, the authors refer to the following works: Rostan, "Sur le Ramollissement du Cerveau;" Turner, "Edin. Med. Chir. Trans.;" Bryant, "Edin. Med. and Surg. Journal;" Storer, "Trans. of a Society for the Improvement of Medical and Surgical Knowledge," vol. iii.; Parry, "on the Pulse;" Andral, "Precis d'Anatomie Pathologique."

Two cases of aneurism of the aorta are also related in this article. As a striking example of the danger that may arise from the neglect of accurate examination in any case, however simple in appearance, the following brief detail is given.

" *Painful Swelling of the Left Lower Extremity. Inflammation of the Saphena Vein. Symptoms of Intermittent Fever.*

"During the month of February 1829, when several cases of ague were in the house, a man named Andrews was admitted, complaining of rigors, followed by a hot and sweating stage, which came on every second day: these, he stated, had been preceded by continued fever.

"Considering the case as an example of tertian ague, we ordered the exhibition of sulphate of quinine in six-grain doses daily. In a few days the type of the fever was changed to that of quotidian, but still, during the intervals, the patient was nearly free from fever. We now made a more accurate examination, and found that the left leg and thigh were extremely painful and swollen, a circumstance which the patient had concealed. Any attempt to extend the limb produced intolerable pain, chiefly referrible to the ham and calf of the leg. The limb was extremely tender on pressure, particularly along the course of the saphena vein, which in its whole extent could be felt like a hard cord. No change could be observed in the temperature of the limb.

"The opposite leg appeared healthy. The saphena was indurated, but not painful on pressure.

"We now omitted the quinine, applied leeches freely to the affected limb, and at the same time exhibited calomel and opium. This treatment proved successful, and the patient was discharged in three weeks with the perfect use of the limb. The saphena having been restored to its original state, that of the opposite side, however, remaining unchanged." (P. 28.)

The intermittent fever in this case may, the authors think, be compared to that depending on urinary disease. There is a point of irritation in the system, which appears to produce the rigors, and to cure the fever we must remove the exciting cause. Our own experience has furnished us with many proofs that these symptoms of intermittent fever, when arising from a local cause, are not to be removed by the same mode of treatment which would subdue pure intermittent fever.

"In this case, as in that of urinary intermittent, the exhibition of bark aggravated the symptoms, and we have had several opportunities of observing that this symptomatic intermittent, although it be reciprocally cause and effect, is exasperated by the above treatment. We have seen it in a lady who had lately been confined, in whom there was a tendency to the formation of mammary abscess. Quinine was exhibited in large doses for several days, and great aggravation of the symptoms of intermittent followed.* In a case of phlegmasia dolens lately treated in the hospital, the woman had daily rigors, followed by a hot and sweating stage. In both these cases the treatment which proved successful was local bleeding and the use of draughts repeated daily, consisting of the ammoniated tincture of valerian, opium, and sulphuric ether. Indeed, in the last-mentioned case, whenever this medicine was omitted, the rigors returned; this happened three or four times. The patient ultimately recovered." (P. 34.)

An accurate observation of numerous cases, both of phlegmasia dolens occurring after delivery, and of painful swelling of the extremities, appearing during or after fever, has satisfied the authors of the pathological identity of the two diseases. In both, œdema occurs, unattended by redness, but accompanied by increase of heat, with great tenderness and pain, and followed for a considerable time by impaired motion of the limb.

"In both diseases the swelling and other symptoms are frequently not confined to any one portion of the extremity, but extend uniformly over the leg and thigh. In both diseases, however, we have also often observed, that the pain, heat, and swelling, occupied

* "In another patient we observed well-marked tertian ague supervene during the administration of large doses of sulphate of quinine. In a case of arthritic rheumatism in a person previously healthy, an imprudent attempt at curing the intermittent by still further increasing the dose of sulphate of quinine, induced a fatal pneumonia: in this case more sulphate of quinine had been exhibited antecedent to the appearance of the tertian fever than would have been sufficient to cure three ordinary agues."

particular parts of the limb, while the rest was comparatively free from disease. Thus in some cases a portion of the thigh was intensely engaged, while the leg and foot remained free, and after some days the diseased action seemed to change its place, and successively attacked the other portions of the limb, without, however, any precise order in the mode of succession. In consequence of this, our treatment has been directed to different portions of the limb, according to the situation of the disease; and we constantly found that the degree of swelling in the part attacked was proportioned to the accompanying heat, pain, and tenderness. In some cases we have observed this affection to be attended by a cordy and painful state of the saphena vein, proving that it participated in the disease; but as this state of the vein, where it did occur, was in some cases subsequent to the disease of the other parts of the limb; and as in the majority of our cases of phlegmasia dolens, and in the painful swelling of the extremity after fever in the male and female subject, no such affection of the saphena occurred, we think that the latter cannot in justice be considered as the cause of the disease. The occasional occurrence of the swelling in the inferior portion of the limb in the first instance, and its erratic nature, militate against the idea that the disease proceeds from an affection of the large venous trunks; and in two instances we have seen the disease desert its original seat, and concentrate itself in the knee joint, producing obstinate inflammation of the part, which in one case, that of a male after fever, terminated in anchylosis, and in the other, that of a female who laboured under true phlegmasia dolens after delivery, the same unfortunate result was with difficulty arrested.

"As the latter occurrence, and many obvious considerations, leave little doubt concerning the inflammatory nature of the disease, it remains to be considered what are the parts engaged. To us it would appear that the subcutaneous cellular tissue is primarily affected, sometimes generally, at others partially. It is not unusual to meet with cases either of general or local anasarca evidently of an inflammatory origin, accompanied by pain and heat, but unattended by redness. The external and vascular layer of the corium remaining uninflamed, will account for the absence of redness in this disease, as well as in the inflammatory anasarca." (P. 35.)

The treatment recommended in such cases, during the acute stage of the disease, consists of local antiphlogistic means persevered in with assiduity; the use of stupes; and, after the disease has continued for some time, the liberal use of narcotics. When more than usually obstinate, these means must be accompanied by bandaging, tonics, and diuretics, and, when the knee is attacked, frequent leeching, blistering, and the use of the inclined plane. The utility of salivation is considered doubtful.

"In one case we obtained no advantage from the liberal exhibition of iodine. We have been informed by an experienced practitioner, that in several cases of phlegmasia dolens, he has observed marked advantage from the repeated application of blisters to the affected limb; a fact evidently not inconsistent with our view of the pathology of the disease." (P. 39.)

Another case is related, which strongly corroborates the opinion maintained by Drs. R. Lee, D. Davis, Tommasini, &c., that phlegmasia alba dolens is in reality owing to phlebitis. An interesting case of disease of the lymphatics is also given.

Dr. Graves and Dr. Stokes enter at considerable length into the description of the various diseases of the respiratory organs, and of the abdominal viscera, which fell under their notice in the Meath hospital, during the session of 1828 and 1829. Many of the cases related in this part of their paper are very valuable, and the therapeutical and pathological remarks appended to them well deserve the attention of the profession.

The next article, by Professor Colles, is entitled "*Practical Observations upon certain Diseases of the Anus and Rectum.*" Although diseases of these parts have of late years much engaged the attention of the profession, it must be admitted that the diagnosis and treatment of such complaints admit of further improvement.

"*Organic Stricture of the Rectum.*" This disease spares neither sex nor rank: it most frequently attacks those who are about the meridian of life; sometimes, however, it afflicts children as early as the seventh or eighth year of their age. Dr. Colles has not met with any instance where it attacked a person at or beyond sixty years of age.

"In some few cases, the patient appears to be aware of the moment of the first attack of this disease; for he tells us, that without any previous illness, the bowels at a certain period suddenly became costive; that for the purpose of relieving them he took large and repeated doses of physic for three, four, or five successive days; that at length his bowels suddenly gave way, and a very severe purging took place, which having continued for a day or two, was then succeeded by those symptoms which attend the disease when fully formed.

"Many patients, however, cannot give any account of the first approach of this disease; they merely state that they have been for many weeks, months, or years, subject to it; that the symptoms from the commencement were pretty much the same as those they now labour under, but perhaps not quite so severe and urgent.

"When organic stricture is fully formed, (by questioning him,) we learn from the patient, that in the course of each day he has many and sudden calls to stool; that at each of these he is obliged to strain very much, and that the straining, which is not followed by any severe pain, produces a discharge of not more than a table-spoonful of mucus, which is sometimes streaked with blood, and very rarely mixed with a small quantity of fæces; that these evacuations are generally attended with a copious discharge of wind, and that, as soon as the evacuation has taken place, he feels free from pain or uneasiness; the remission however is of short duration, as he is soon again compelled to undergo the same unavailing distress. The number of these discharges are seldom less than from seven to twelve during each day and night; they do not take place at regular intervals, but generally a considerable number occur in quick succession, and then are followed by a pretty long interval of ease. The greater number of these evacuations are devoid of fæces; a feculant stool is passed perhaps once in two or three days; the fæces are then found passed in short pieces and of very reduced dimensions, not larger than a full-sized catheter, and in quantity not equal to what is passed in an ordinary evacuation by a person in health, yet after each feculent stool the patient feels much relieved.

"There is not the slightest prolapsus ani with any of these evacuations.

"The bladder is in some cases slightly affected; the patient then complains of a little difficulty or delay in passing his urine.

"In some cases a fistulous opening forms in the nates or the perinæum, which will admit a probe to pass into the rectum; it yields a moderate quantity of healthy pus. The fistula undergoes little or no change from the time of its first appearance, even until the death of the patient. In some cases, especially in females, I have known the number of these fistulous openings to amount to twelve or twenty. The majority of cases of stricture of the rectum, however, are unattended by fistulæ.

"Although this state of daily suffering proceeds with the most unvarying regularity, not only for weeks and months, but even for many years, yet the constitution of the patient does not seem to sympathise in the slightest degree for a long time; not only do his colour and appearance proclaim the enjoyment of good general health, but even the most strict examination cannot discover in any of the functions the slightest deviation from health, except those above mentioned.

"After a lapse of time, however, which is very different in different cases, a peculiar paleness of countenance and wasting of flesh announce an inroad on the constitution: those symptoms are soon followed by night sweats, and now the patient complains of uneasiness about the sigmoid flexure of the colon, which soon extends along the left colon.

"In this stage of the disease, it too frequently happens in females that a communication is formed between the rectum and vagina, which causes the greater part of the entire of the fæces to pass through the latter. In men, but more rarely, a similar communication is established between the rectum and bladder.

"As the disease draws towards a close, the patient begins to suffer much more serious distress from the state of the rectum; for, in addition to the frequent unavailing efforts to discharge the bowels, he is troubled, on the slightest exertion, such as coughing, sneezing, or voiding urine, with an involuntary discharge of a thin brownish fluid, of a muco-purulent nature. After some time, this peculiar discharge comes away in the morning, the moment the patient rises from his bed. In the last stage of the disease, this discharge comes away unceasingly, without the consciousness of the patient, and the fæces will not pass unless in a perfectly liquid state.

"This aggravation of local disease is accompanied by a corresponding decline in the general health: his appetite fails, thirst becomes very urgent, emaciation proceeds rapidly, hectic fever becomes fully formed, though more marked by profuse night-sweats than by mid-day or evening exacerbations; and now the patient, extenuated to the last degree, seems to be carried off as much by the exhausted state of his constitution as by the torments of the local disease.

"Sometimes, in the advanced state of this disease, the patient is seized with symptoms of peritoneal inflammation, which puts a speedy termination to his sufferings, and he is suddenly carried off. In such cases, on examination after death, we discover that the process of ulceration had opened the intestine immediately above the stricture, and that through this opening a portion of fæces had passed into the cavity of the abdomen.

"In some very few patients, the hectic fever seemed to have been arrested by the warmth of summer weather, but only to run the remainder of its course with unusual precipitancy on the approach of winter." (P. 132.)

Among a considerable number of patients afflicted with this disease, Dr. C. has had an opportunity, in two instances only, of meeting with it in its incipient state. In both of these the patient complained of different symptoms of irritation of the rectum, frequent stools, discharges mixed with mucus, and certain feelings of uneasiness. On examination by the finger, a thickening and slight projection of the gut were felt at a small spot on one side. These morbid alterations spread gradually round the entire of the canal, and extended along it only to a small distance; but until the morbid derangement of structure had almost entirely performed the circle of the intestine, the patient did not exhibit those symptoms which Dr. Colles considers as the common and inseparable attendants on stricture of the rectum. Our opinion of the nature of the disease must be confirmed by manual examination, however well marked the symptoms may be.

"Proceeding to make this examination, we often observe, at the orifice of the anus, the following appearance, which is indeed almost always present when the disease is seated near to the external sphincter, namely, at each side of the anus a small projection, which, on its external surface, appears as a mere elongation and thickening of the skin, but internally presents a moist surface, not exactly like the lining membrane of the gut, nor yet can we say that it is ulcerated: these two projections lie close together below and divaricate above, presenting a resemblance to the mouth of an ewer. Whenever this external appearance exists, I feel almost certain of finding a stricture of the rectum, before the finger is pushed as far as the second joint into the gut. In some cases, however, this external mark has not been present.

"When the stricture is situated pretty high up, the portion of gut interposed between it and the anus is found to be in a perfectly healthy state; but, when the finger arrives at the stricture, it is arrested by the narrowness of the canal, which will barely admit the point of it: if now a slight degree of force, combined with a boring motion, be employed, the finger may be pushed through the thickened and indurated part, and will then (as well as its benumbed condition permits it to feel,) find that the gut just above the stricture is in a very healthy state. The extent of the stricture, however, is very variable: sometimes it is little more than a mere ring, but at other times it extends along the canal as high as the finger can reach.

"I have not yet with met with any instance in which the intestine was strictured, only by means of bands thrown across its canal: such cases, I presume, must be very rare." (P. 137.)

In a few instances the stricture has been found seated so high in the gut, that it could only be barely touched with the point of the finger, until the patient was desired to "force down," and then a satisfactory examination of it could be made. With ordinary attention, this disease cannot be confounded with any other; but as it presents some symptoms in common with other diseases both of the rectum and the adjoining organs, Dr. Colles notices the leading distinctive characters between stricture of the rectum and these other affections, as cancer of the rectum, a rare form of scirrhus of the uterus and vagina, which gives rise to a train of symptoms not unlike those attending stricture of the rectum; ulcer in the rectum; and tumors in the pelvis, which may compress the rectum so as to narrow and materially obstruct the canal. Dr. Colles has "nothing cheering to offer on the treatment" of organic stricture of the rectum. In this very candid expression of regret, we believe all other surgeons must participate, who have had to contend with this formidable malady. Mercury, arsenic, cicuta, and iron, have been tried in vain.

"Large quantities of mucilage appear to give most relief. Bluepill, combined with a double quantity of Dover's powder, has also occasionally afforded much temporary alleviation." (P. 143.)

The treatment by bougie, usually recommended in this disease, appears well calculated to alleviate the sufferings of the patient; but Dr. Colles feels confident that a perfect cure of the organic stricture of the rectum has not been effected by any plan of treatment hitherto employed, and he therefore very properly urges his surgical brethren to turn their attention to the subject, and earnestly to endeavour to discover some other means of relieving so distressing a disease. Dr. C. cannot admit the existence "of what is termed the spasmodic stricture of the rectum."

Dr. Colles next offers some observations upon vascular tumors of the rectum.

"The opinions of the profession are divided as to the best mode of removing these tumors, some preferring the ligature, others excision: I am decidedly in favor of the latter, for this reason, that tetanus, the consequence of which is to be dreaded in the former mode of proceeding, cannot by any means be foreseen or guarded against; whereas hemorrhage, which alone is to be feared in the operation by excision, may be prevented, and can most certainly be suppressed." (P. 152.)

Upon this subject we cannot altogether agree with the author. We have often applied the ligature to such tumors ourselves, and we have more frequently seen it applied by others. We never saw any mischief arise from the practice. The patient suffers, indeed, considerable pain; but this necessary consequence of the operation may, in most cases, be greatly, if not entirely, controlled by the judicious exhibition of opium. We fear excision, not from any personal experience of its danger, but from the fatal result which has followed from hemorrhage when this practice has been adopted by the ablest surgeons. We doubt the "certainty" with which the hemorrhage can be suppressed. We do not intend to deny that some hazard may attend the application of the ligature to these tumors, but we believe it is not so great as that which is to be feared from hemorrhage after the operation of excision. With respect to the mode of applying the ligature, we may refer to the remarks we have made in our notice of Mr. Castle's "Manual of Surgery," in the present number. To guard against the danger of hemorrhage, of which Dr. Colles is much less apprehensive than we are inclined to be, the following precautions are suggested:

"I take care not to prolong my incision higher on the bowel than what I conceive will, when replaced, lie within the circle of the sphincter; for, if we cut the gut higher up, this part, when returned, may bleed freely from not having any surface closely opposed to it. Besides, we know that, by cutting higher up, we are in danger of cutting the trunk of the vessel, instead of confining our incision to the tumor, which is composed solely by the convolutions of its very minute branches.

"I have said that, if hemorrhage should follow this operation, it can most certainly be arrested. This I have done satisfactorily in a case where the patient had suffered for nearly twelve hours by bleeding into the gut, from which it was at intervals shot forth involuntarily, with forcing and straining: many and unavailing were the methods employed by three or four surgeons and myself, until at length I bethought me of Petit's method of stopping an hemorrhage from the rectum. In this case I made use of sponge, instead of the lint recommended by that author.

"I should be afraid to adopt Mr. Hey's method of cutting away all the protruding tumors, together with the skin at the verge of the anus, lest the patient should afterwards experience that distress which a too contracted state of this outlet must occasion; for in one case where, for the purpose of extirpating warts, a ring of skin at the verge of the anus had been cut away along with these excrescences, the condition of the patient was rendered truly miserable." (P. 153.)

Dr. C. remarks, that it may be satisfactory to timid patients to learn that this disease of vascular tumors may be rendered very mild, if not ultimately cured, without an operation.

"By causing the patient to inject four or five ounces of an injection, consisting of two grains of sulphate of zinc to each ounce of water, every night going to bed, and making him retain it during the night, I have known the disease so much alleviated, that patients have flattered themselves they had obtained a perfect cure. I have known some of them, though not living in the most regular manner, yet enabled to enjoy all the sports of the field without inconvenience. I fancy that this plan of treatment has succeeded best when begun immediately after one of the painful fits of the disease. I have known many of these patients much benefited by smearing, after each evacuation, the protruded parts with a liniment of Ol. Oliv. ℥ii. and Plumbi Subacetat. Liquor ʒi." (P. 155.)

Dr. Colles concludes his paper with a few cursory remarks upon ulcer of the rectum.

The next communication is by Mr. Houston: "*Observations on the Mucous Membrane of the Rectum.*" The principal object is to show that, in the natural state, the tube of the gut does not form, as is usually conceived, one smooth uninterrupted passage, devoid of any obstacles that might impede the entrance of bougies; but that, on the contrary, it is made uneven in several places by certain valvular projections of its internal membrane, which standing across the passage, must frequently render the introduction of such instruments a matter of considerable difficulty. Plates are given to illustrate the valvular projection described, and we must therefore refer to the work.

Mr. Beatty next contributes a paper upon "*Aneurism of the Abdominal Aorta*," of which the purport is to assist future observers in the detection of this disease, the symptoms of which are frequently uncertain and obscure.

Two cases of "*Aneurism successfully treated by Ligature*," are related by Mr. Porter. In one there was aneurism of the left subclavian, in the other of the right carotid artery. Both these large arteries were tied with the single round ligature; a mode of treatment which, according to Mr. P.'s experience, is more successful than any other contrivance for arresting the current of blood, whether by the *presse artère* or otherwise. The carotid was tied within a quarter of an inch of its origin from the innominata; a circumstance heretofore considered as likely to interfere with the success of the case, by preventing the formation of an internal coagulum. The very trifling disturbance of constitution that followed on the tying of so important a vessel as the subclavian artery, is also pointed out as an interesting fact; the patient never having a single symptom that could occasion anxiety as to the result, until the occurrence of inflammation and suppuration of the sac. Both patients recovered, notwithstanding suppuration, an event which has always been considered as pregnant with the most dangerous consequences. Indeed, in the case of carotid aneurism, the woman's life was three times in such imminent peril, as almost to preclude hope; but in the other instance the patient recovered with as little trouble, and perhaps with more rapidity, than if it had been a case of common aneurism.

"William Austin, æt. forty, by occupation an hostler or helper about livery stables, applied to me on the 25th June, 1829, complaining of pain in the left shoulder, arm, and hand, together with a distressing sense of numbness, contraction of the fingers, and œdema of the lower portion of the limb. The pain in the arm and hand was so excessive as entirely to prevent him following his usual employment, and occasionally to deprive him of sleep. He had a firm pulsating tumor below the clavicle, in that part corresponding to the division between the pectoralis major and deltoid muscles, about the size of the half of a hen's egg, becoming smaller on being pressed, and losing its pulsation, and almost disappearing when the artery is compressed above the clavicle. He has no recollection of having ever sustained any injury in that part, neither had the tumor been observed by him until eight or nine days since. Even on his examination he could scarcely be persuaded that the tumor, in which he felt no pain, could be the cause of, or connected with, the uneasiness and numbness of the arm.

"He was immediately admitted into the Meath Hospital, and confined to bed, with orders to maintain a state of perfect quietude: cold was applied to the tumor; saline purgatives administered every day, and venesection on the 27th, to the extent of fourteen ounces. Notwithstanding this treatment, the tumor increased with great rapidity, extending outwards towards the axilla, and slightly displacing the clavicle upwards, yet the derangement of this bone was so trifling as not to be observable without close examination. On the 29th the tumor had increased to more than double its original size, and as its further growth might, by pushing the clavicle upwards, derange the relative anatomy of the part, and add materially to the difficulty of the operation, it was determined in consultation that the subclavian artery should be tied on the following day." (P. 198.)

Mr. Porter states that there are but two situations in which the subclavian artery on the left side can be taken up before it reaches the first rib, namely, whilst it is yet between the scaleni muscles, and after it has passed the outer edge of the anterior scalenus.

"The former of these operations has been (as far as I know,) only performed by Baron Dupuytren, in a case, the particulars of which are detailed by M. Marx, in the 2d No. of the Répertoire générale d'Anatomie, and the advantages attendant on the choice of this situation are stated to be, 1st, the facility of cutting down on and exposing the artery here: 2d, the impossibility of interfering with or injuring the great subclavian vein: 3d, the great nerves being thus placed in a comparative state of safety. But in M. Marx's report, although otherwise carefully drawn up, I find no mention whatever made of the phrenic nerve, which, although subject to occasional trifling irregularity of situation, is always so placed in front of the anterior scalenus that it could only be avoided in a division of this muscle by a protracted and tedious dissection, and perhaps not always, even with this precaution: and it may be here remarked, that in the sketch of the operation which accompanies the paper, no trace of the nerve is to be observed at all, an omission which, occurring under the eye of so excellent an anatomist, appears inexplicable. Again, I find no mention made of the thoracic duct, which lying just at the internal edge of the scalenus, must have been fearfully endangered. It is true, that as the progress of the case presented no untoward symptom, and, as it eventually terminated in the recovery of the patient, there is every reason for believing that neither of these very important parts were injured: yet, as the mere knowledge of the existence of such difficulties must add to the embarrassment of the operator, and their avoidance cause both trouble and delay, they furnish very good grounds for selecting any other possible situation in which the artery may be secured. Add to these, that generally there are three large branches given off by the subclavian whilst it is between the scaleni muscles, so that even in the dead subject it is difficult to find space sufficient to permit a needle being turned round the trunk without injuring one or other of them; and that the first dorsal nerve, from its situation behind the artery, would be extremely liable to be included within the ligature. These reasons will be sufficient to show why this situation was not preferred in the present instance, and why it should never be chosen, unless the aneurismal tumor be so placed as to prevent the exposure of the vessel after it has passed the scaleni, where it has hitherto been so frequently tied with success." (P. 200.)

The operation was performed as follows:

"The patient being placed on a table, his head supported upon a pillow, and thrown back as far as possible, whilst his shoulder was depressed by an assistant, an incision was made along the edge of the clavicle, commencing midway between the centre of the sternum, and the acromion process, and carried outwards to the extent of two inches and a half. From the sternal extremity of this incision another was carried along the line of the external edge of the scalenus, and the triangular flap thus formed was dissected back, exposing the fascia, the external jugular vein, and a plexus of small veins communicating with it. In carrying the incisions deeper, a small vein was cut off close to the trunk of the jugular, which poured out so much blood as to obscure the parts, and to render the application of a temporary ligature necessary. The perpendicular incision was carried no deeper than to expose the fibres of the scalenus muscle, after which the remainder of the operation was completed with the handle of the knife and the finger. The detachment of the cellular tissue in this manner was easily accomplished, but required a good deal of time, in consequence of the narrowness of the wound in its deepest part, the entire breadth of which extended only from the external edge of the scalenus to the trunk of the jugular vein, a space of not more than three quarters of an inch. Had I deemed it advisable to divide this vessel, the time occupied in the operation must have been greatly lessened, but in that case the patient either should have lost a very considerable quantity of blood, or have been exposed to the hazard attendant on the application of a ligature around the vein, a risk which I consider by no means of a trifling nature, and which ought to be avoided at any sacrifice of time whatever.

"On exposing the omo-hyoideus muscle, a very large artery, (probably the transversalis humeri,) with its accompanying vein, was seen to traverse the middle of the perpendicular incision, in a situation where it might easily have been wounded, had the edge of the knife been used incautiously. The large nerves next came into view, and one of them receiving an impulse from the artery, was for a moment mistaken for it; but on applying pressure, and finding that the circulation in the limb was not thereby commanded, that idea was abandoned, and the vessel sought for still more deeply. A

few more touches of the handle of the knife were sufficient to lay it bare:* the needle was introduced from above, and the ligature thrown round it with the greatest facility. It was then tied firmly, and the pulsation in the aneurism and in the radial artery ceased instantaneously. One end of the cord was cut off close to the knot, the other left hanging out of the wound, the edges of which were brought together, one stitch only being applied to the angle of the flap.

"The patient lost not more than three ounces of blood during the operation, and walked from the theatre to his bed in an adjoining ward.

"Immediately after the operation he experienced some uneasiness in the left arm, with a peculiarly disagreeable sensation, which he could not describe; but, on the limb being wrapped in flannel, he expressed himself quite relieved, and from that moment remained perfectly tranquil, without any symptom that could excite the smallest apprehension. No perceptible alteration of temperature could be observed at any time in the arm: both limbs were carefully examined by the thermometer at different times, but the instrument never indicated any difference between them in this respect.

"On the second day after the operation, the veins on the back of the hand were large: the radial artery evidently full of blood, and feeling like a soft cord under the finger. On the tenth day, a slight thrill was perceived in the vessel, probably communicated by the current of blood through it, and many who examined the patient on this and subsequent days, considered that a distinct pulsation could be felt synchronous with the action of the heart, but of course extremely weak. However, I have never been able to satisfy myself that such pulsation actually existed. On the morning of the seventeenth day, the ligature came away without the loss of a single drop of blood. The suppuration was small in quantity, and of the healthiest character. The aneurismal tumor was greatly diminished in size, and its edges scarcely defined." (P. 202.)

No constitutional symptom occurred after the operation, "worth recording." The patient never suffered any inconve-

* "The instrument used in this as well as in the following case, was made by my ingenious friend, Mr. F. l'Estrange, and is, as far as I know, of his invention, although by some it is believed to have been first used in America. It is of the usual shape, but is furnished with two eyes, and the lower portion containing the eyes is removeable from the shaft by means of a screw. One of the eyes carries the ligature, and the other is destined to be laid hold on by a small hook after the instrument has been passed under the vessel. When thus secured, the shaft of the needle is to be unscrewed, and the lesser portion with the ligature is easily drawn up. I have given a sketch of this needle, in order that its construction may be perfectly understood, because I believe that, by means of it, a ligature may be thrown round any artery, no matter how deeply situated."

nience, except from confinement to bed, and the restrictions on his appetite which were deemed necessary. In less than four months afterwards, Mr. Porter saw the man actively engaged in his former occupation, as assistant in a stable, and he then said he was as well able to earn a subsistence as at any former period of his life.*

The case of carotid aneurism is interesting, on account of the narrow escape the woman had after the operation: the skilful treatment and zealous attention of Mr. Porter overcame, however, all the difficulties of the case, great as they were, and the life of the patient was not only saved, but she was also again enabled to resume her occupation as a servant.

We shall take an early opportunity of giving an analysis of the remaining communications in this volume, many of which are highly creditable to the contributors.

---

*Surgical Observations on the more important Diseases of the Mucous Canals of the Body: being a Second Edition of the Author's Treatise on Stricture of the Urethra. To which are added, Practical Observations on Contraction of the Œsophagus and Rectum; an Essay on the Diagnosis of Hernial and other Tumors in the Groin; with Remarks on Tracheotomy, as connected with the Treatment of Chronic Laryngitis.* By George Macilwain, Member of the Royal College of Surgeons, &c.—8vo. pp. 362. Longman and Co.: London, 1830.

This is a very instructive volume; and one of its chief merits is, that the author of it has no peculiar practice to recommend; for we generally find, when either a surgeon or a physician is the parent of a new mode of treatment, that it is pushed beyond all reasonable and useful bounds, and is made to apply to cases in which it is either useless or hurtful. Mr. Macilwain truly enough observes, that any surgeon acquainted with the works on stricture of the urethra, will perceive that the authors generally recommend one kind of treatment, to the virtual exclusion of all others. But the variety which exists in the nature of strictures does not admit of the exclusive adoption of any one mode of practice; and Mr. M. has therefore endeavoured, by attentive observation, to ascertain the particular class of cases to which one or other kind of treatment was

* "It may be proper to mention, that this patient received a fall on the shoulder soon after, which affected the arm with paralysis for some weeks. He has, however, since recovered, and is at this moment employed as a helper in a stable, but certainly he is not equal to severe or continued exertion."

especially applicable. His object is not, then, the promulgation of any thing new, but the proper application of that which is already known.

As we accidentally omitted to notice the first edition of this work, we shall enter at some length into the consideration of the present improved and enlarged volume.

The first chapter treats of strictures of the urethra in general, of the different situations of strictures, the causes giving rise to them, and the kind and degree of alterations of structure by which they are accompanied. Every part of the urethra, excepting its prostatic portion, is liable to become contracted by this disease, but it occurs more frequently in some situations than others. Strictures are more frequent in the membranous portion, and especially in that part of it which is immediately posterior to the bulb; they also very commonly occur immediately anterior to the commencement of the corpus spongiosum. The external orifice is but seldom the seat of stricture.

"It is stated in some books, that strictures frequently happen at the bulb: if by this we are to understand that part of the urethra actually covered by the posterior extremity of the corpus spongiosum, I confess my experience has led me to think differently. That the urethra does become narrower at this part, must be admitted; but by no means, I think, so frequently as has been represented." (P. 3.)

The form and extent of strictures vary considerably. The canal may be simply narrowed, as if a ligature had been thrown around it; and "there are instances in which the whole tube, from its membranous portion forwards, has become lessened in its diameter." Sometimes there is a sort of bridle thrown across the urethra, and the impression which in such a case is made on a soft bougie, resembles that which would be produced by drawing a piece of fine twine across its point. The author has met with several cases of this kind. The change which the mucous membrane of the urethra undergoes, consists of a greater or less thickening.

"In some instances it proceeds so far, that no similarity with its healthy condition is discoverable, appearing almost of a cartilaginous texture, whilst in others scarcely any change can be observed; and there are degrees of alteration intermediate between these extremes. Where the contraction is considerable, and the membrane but little altered, there is a thickening produced by deposition exterior to it. The change of structure is generally proportionate with the duration of the complaint; and, if this have been very long, the whole of the urinary organs usually becomes more or less diseased." (P. 5.)

There is no morbid condition of the bladder, ureter, or kidney, which may not co-exist with stricture of the urethra. Any circumstance capable of producing violent or continued irritation in the urethra may produce stricture. There is no cause so frequently referred to, as productive of this disease, as gonorrhœa: but whether it exerts so extensive an influence in originating it as is generally supposed, may, in Mr. Macilwain's opinion, be fairly questioned. The occurrence of stricture from the use of injections is admitted, but only when the latter have been injudiciously employed. The experience of Mr. Hunter shewed that strictures occurred as frequently after gonorrhœa treated without injections as with them: Mr. M. could readily believe much more frequently, and for the following reasons.

"Diligent investigation shows that stricture does not commonly proceed from violent inflammation, but from a sort of irritation, moderate but continued, principally characterised by a morbid increase of sensibility, sometimes attended by discharge. Thus it is found that where stricture can be traced to gonorrhœa, the latter has been neglected, and the irritation, of which the discharge is sufficient evidence, unnecessarily protracted; during which period the patient is liable to sources of excitement which he is wearied with foregoing. No state can be more favorable to the formation of stricture than that to which I have just referred. Injections, therefore, when properly employed, by removing the irritation and discharge, so far from conducing to the occurrence of stricture, become actually indirect preventives of this disease. The consideration of gonorrhœa would lead to a digression: I shall therefore merely mention the principles to which it is necessary to attend in the employment of injections: 1st, they should never be used while there is pain in micturition; 2dly, their quality should never be of such a nature as to produce more than a temporary smarting; 3dly, their strength should be increased as the canal becomes less susceptible; and, lastly, their action should be confined to the seat of the disease." (P. 10.)

Strictures are, it is said, generally preceded by a peculiar state of the canal, denominated irritable urethra: but the urethra may labour for a considerable period under this affection before contraction takes place in any part of it, of which the author has seen repeated examples. The diagnosis between the symptoms which arise from a merely irritable urethra, and positive stricture, is confessedly obscure; but still, in many cases, Mr. M. thinks it sufficient to enable a discriminating practitioner to distinguish the two maladies.

"First, then, with reference to the volume of the stream in micturition. Persons with irritable urethra will occasionally make an exceedingly small stream, yet at other times it shall be of the natural size: now, although in stricture its volume varies considerably at different periods, yet it is never to the same extent; attentive inquiry showing it always to be less than natural. Again, we find also that the diminution has progressively become more considerable, which is not the case in simply irritable urethra. It is by no means uncommon to find patients making water in a full stream after a meal, although previous to this it was exceedingly small, and the urethra so irritable as to give a feeling of strangury; a difference which never happens to the same extent in stricture. Involuntary emissions* are occasionally attendant on stricture, but they are scarcely to be considered as a common symptom; whereas, in simply irritable urethra, nothing can be more frequent: and I am the more confident that this difference exists, from finding that they are sometimes described as a symptom of stricture occurring at an early period of the disease.† (P. 14.)

As far as the author's observation extends, a greater or less disorder of the general health usually accompanies an irritable urethra; whereas, in stricture, it is frequently by no means so easily discoverable. We have often ourselves seen patients with very irritable urethræ, who certainly neither complained of nor evinced any other derangement of health. We should have but little confidence, therefore, in this presumed distinction; it is not, indeed, very positively laid down by Mr. Macilwain: nor have we ever known abscess in perinæo arise from a merely irritable urethra.

Painful enlargements of the testicle, and hydrocele, are occasional attendants on this complaint. One case has occurred to Mr. M., which leads him to think that even organic disease of the testicle may result from an irritable urethra, when its influence is exerted on a disordered constitution. In some cases of irritable urethra, the only treatment that is necessary is change of air and attention to the general health. In general, however, the treatment consists in the introduction of instruments, as advised by Mr. Abernethy. By this means the morbid sensibility of the canal is diminished, by

* "I have seen very many cases tending to establish this point. One morning, whilst this treatise was in course of publication, I had occasion to examine the urethra of a patient who consulted me a few days previously for symptoms which rendered him confident that he had stricture. On his informing me that, since he had employed the measures recommended to subdue the irritation, his stream had been on some occasions as large as natural, I recollected that he had not complained of involuntary emissions. On making an inquiry as to this point, he said that he was very frequently troubled with their occurrence. Examination detected a highly sensible prostatic urethra, but nc stricture."

† "Howship on the Urinary Organs."

habituating it to the mechanical stimulus of an instrument. Before, however, we have recourse to instruments, certain preparatory measures should be adopted, which are described under the treatment of stricture. "We should commence by an instrument calculated to produce the least irritation, and increase this in proportion to the diminishing susceptibility of the urethra." Mr. M. generally commences with No. 10, increasing its size at every subsequent introduction.

In the third chapter, the student and junior practitioner will find some very useful hints respecting the introduction of bougies in general.

The fourth chapter gives a clear and very correct detail of the symptoms of stricture. The author believes that the straining which accompanies stricture not unfrequently gives rise to hernia,* and hence an additional reason why the treatment for the removal of strictures should be commenced at an early stage of the disease. We are not acquainted with any facts which confirm this, to us, novel opinion; but we are not, therefore, inclined to doubt the result of Mr. Macilwain's extensive experience upon the subject. It must be presumed, however, that if hernia be thus produced, there must have existed some previous and very strong disposition to its occurrence. It is important to know that patients have consulted physicians for disorder of their general health, which, from its obstinate nature, and from its subsiding only on the discovery and subsequent removal of stricture, evidently depended on the latter affection.

"I saw, about a year and a half since, a remarkable case of this kind. A man, about thirty-eight years of age, had been admitted in the Finsbury Dispensary, under the care of my respected colleague, Dr. Hancock, with a very much disordered condition of his general health: his secretions were unhealthy, his tongue furred, and he suffered such excruciating pain in the different joints of the body, that he was confined entirely to his bed, not being able to stand. He could obtain no rest at night, although, in addition to various remedies usually employed in rheumatism, he had others especially directed to procure sleep. No benefit whatever resulted from the treatment: at last, his micturition became painful, and attended with a considerable discharge of muco-purulent matter. He now mentioned to Dr. Hancock that he had a stricture, who immediately referred him to me. I found him in a most deplorable condition; for, besides the foregoing symptoms, he now suffered from occasional rigors, which seemed to exasperate the pain in his

* Mr. Lawrence has observed some analogous facts. See his valuable Treatise on Ruptures, p. 18, second edition.

joints, and which were otherwise exceedingly distressing to him. Examination discovered that he had a much strictured urethra, which was put right by means of the silver catheter, assisted during the treatment by three applications of the kali purum. As his urethra became free, his sufferings were rapidly mitigated, and in two months he was quite well." (P. 48.)

The next chapter contains "General Observations on the Treatment of Strictures." The principles on which the restoration of the canal to its natural condition has been attempted, have varied considerably. In general, the object has been either to effect a mechanical but gradual dilatation of the contracted portion; to remove the impediments, by the application of substances, the precise nature of whose action is somewhat undeterminable; or, lastly, to destroy them by caustic. Mr. Macilwain prefers bougies to the instruments called dilators, but he confesses that he has not been in the habit of using the latter. His reasons, however, for preferring the former appear to us ingenious and satisfactory. It is fairly argued, that a very little consideration of those changes which constitute disease, or those by which organs are restored to their healthy condition, will convince us that neither the one nor the other can be effected suddenly; and that the safety with which the latter is effected, is generally, more or less, in proportion as its accomplishment is gradual. This remark is equally applicable to the diseases of other parts, as it is to those of the urethra. The practice of endeavouring to overcome an impermeable stricture by thrusting a conical catheter onwards, is very properly deprecated, as fraught with danger, and never therefore admissible. We trust, indeed, that this practice is now entirely given up by English surgeons, although its occasional adoption in other countries is still to be lamented. "I would recommend," says Mr. M. "those who are disposed to follow this practice, previously to inspect the different preparations in this town, shewing the frightful deviations which have occurred in the passage of instruments from the route intended." Let it be remembered, too, that the most accurate knowledge we can possess of the anatomy of the parts cannot enable us to ascertain that we are employing force in the right direction. If we were justified, we could a "horrible tale unfold," in which a surgeon did force his instrument into the bladder in this manner: the patient died, and, upon dissection, it was shown that the intended track had been *somewhat* deviated from, and that the parts had been *somewhat* lacerated.

"In cases where no instrument can be passed through a stricture, by the employment of caustic or other means, it is better to cut down to, and divide, the stricture."* Catgut bougies are never employed by the author. "We have no control over the direction of the point of these instruments, any more than we have over those of elastic gum, when used without a wire in their centre." The common wax-cloth bougie is said to be of little use, except for taking the impression of a stricture, for which purpose it is well calculated: for any other, the elastic gum is preferable, because it is smoother than the common wax-cloth, and glides more easily through the urethra. Of all the metallic bougies, the silver catheter, in Mr. M.'s opinion, has great claims to our preference. The following extract conveys the author's views of the caustic applications.

"Impressed as I am with the various occasional inconveniences attendant on the use of the argenti nitras, even in the most skilful hands; and believing, as I do, that in general the kali purum is an equally effectual, and certainly a safer application, I feel obliged to admit, that there are a few cases in which the use of the argenti nitras is to be preferred, and which will be described in their proper place." (P. 62.)

In the treatment of ordinary stricture, we have two objects to attain: a restoration of the canal to its natural diameter; and, secondly, security from the intervention of untoward symptoms during the treatment. The practitioner must constantly bear in mind, that stricture can only be overcome or removed in a gradual manner. If the instrument be introduced too often, or its size be increased too rapidly, the urethra will become so irritable that some time must elapse before the treatment can be proceeded in. "As a general rule, it may be laid down that, as long as the improvement is progressive, the surgeon and the patient should be satisfied.

Mr. Macilwain very candidly, and we are sure very truly, admits, that it is often extremely difficult, and in some cases impossible, to prevent the recurrence of stricture.

"Practitioners have usually attributed the return of stricture to some defect in the treatment; and that many cases admit of this explanation, is sufficiently obvious, but I by no means believe that it applies so extensively as has been supposed. However perfectly we may restore the canal to its healthy condition, it is not in our power to remove the indiosyncrasy which occasionally disposes patients to the attack of stricture, much less to prevent them from exposing themselves to new sources of excitement. Even during the treatment, when the more distressing symptoms are relieved, it

* We have recently seen this operation performed in different cases by Mr. Mayo, with great dexterity and success. No surgeon, however, should attempt it, whose hand is not well practised in operative surgery, and who does not possess the most correct anatomical knowledge of the parts.—Rev.

is often extremely difficult to induce individuals to adhere strictly to the rules prescribed. As it is my object faithfully to give opinions warranted by practice, I must here candidly acknowledge, that I know of no treatment which will uniformly prevent the return of strictures, although in many cases it may certainly be accomplished, provided the patient avoid all sources which have an obvious influence in their production." (P. 70.)

The constitutional treatment which is necessary, where the urethra is very irritable, before we begin the use of bougies, is briefly and clearly described. Where there is evidence of greater irritation than usual, the author has found great advantage from suppositories (Opii gr. ii., Hyoscyami gr. v.) placed in the rectum the night before the examination of the urethra in the morning.

"I cannot, indeed, speak too highly of suppositories, since I have several times proved their efficacy by means especially directed to that purpose. We cannot, indeed, expect that their beneficial effects should be permanent; but I have frequently known a patient, whose rest was completely destroyed by his calls to make water, sleep the whole night without being disturbed, after the application of a suppository; and, however temporary the tranquillity thus induced may be, it is always of the greatest consequence, by the facility it affords us of examining the canal." (P. 74.)

Our choice of the size of the instrument should be regulated by the volume of the stream of urine which the patient passes. Mr. M. generally employs one a size larger than the stream is represented to be, when its volume is most considerable. It is very desirable that we should gain a knowledge of the state of the whole urethra as early as possible; for, should we find that there is more than one stricture, the progress of the case will be rendered much quicker by directing our attention especially to that which is nearest the bladder.

"If the canal be exceedingly small at the seat of stricture, the silver catheter is to be preferred to the elastic-gum bougie; for we have no control over the point of a small instrument of the latter description. I cannot too strongly recommend the greatest care in the use of the small silver catheter, since it is especially calculated to do mischief, if more force be used than is sufficient to keep it fairly in contact with the obstacle. Where, indeed, the opening through the stricture is sufficiently large to allow of our employing an elastic-gum instrument, there is considerable advantage in resorting to the silver catheter at the latter period of the case, for reasons which have been before mentioned in relation to irritable urethra." (P. 77.)

When the catheter or other instrument has been passed into the bladder, it is right to leave it there for a few seconds only at first. The size of the instrument should be gradually increased at each subsequent introduction. At first it will rarely be advisable to repeat the use of the instrument oftener than once a week. It sometimes happens that, having dilated the strictured part to a given point, we are unable, notwithstanding the absence of any particular irritation, to pass an instrument of increased size at the next visit. In this case we may, by passing it to the stricture at two or three subsequent periods, be eventually enabled to conduct it through the canal into the bladder.

"The pressure thus made on the stricture may probably induce ulceration; it is objectionable, however, from its uniformly rendering the stricture so exceedingly sensitive; and, as considerable delay is occasioned by this mode of proceeding, I prefer, when I cannot increase the size of the catheter, employing the kali purum. I am informed that many practitioners have altogether relinquished the employment of this remedy, and I can only attribute it to their having expected more from it than it is capable of accomplishing, or to its having been incautiously applied. The kali has certainly appeared to me to be unnecessary in some instances, and (in the extent that we are warranted in employing so powerful a caustic,) inefficient in others; yet again, there are some cases in which it is a very useful assistant, and a few in which it claims decided preference. In the use of the kali, I follow the directions of Mr. Whately, with the exceptions mentioned in the fifth chapter. In all the cases (and the number is very considerable) I have scarcely met with any unfavorable consequences resulting from it. It generally increases the tenderness of the part for a few hours, perhaps a day; and I have seen, though very rarely, pain in micturition for two or three days succeed to its application, but this is the worst consequence that I have ever witnessed.* The particular case in which this remedy is to be preferred to all others, will be mentioned in its proper place. Under the circumstances before mentioned, one or two applications of the kali will enable us to proceed with the treatment, and often render the urethra capable of admitting an instrument a size or two larger than that which, before its application, we failed to introduce. The period of treatment is thus shortened, and the patient generally suffers much less from the kali purum than from the repeated pressure of the silver catheter. The caustic should now, of course, be laid aside, and the original plan persevered in." (P. 79.)

When we are enabled to introduce the full-sized instrument,

* "I have met with one case in which the application of kali produced absolute retention of urine. This, however, was quickly relieved by warm bathing and aperients, and the stricture then admitted an instrument three sizes larger than the last attempted to be used. The patient got quite well, without any further interruption taking place in the successive introduction of instruments of increasing diameter."

it should be passed occasionally for a week or two. The patient should be guarded in his future mode of living. In severe cases, the urethra should be examined "once a year," because, if any disposition to stricture recur, it may with facility be removed.

In the seventh chapter, those cases are pointed out to which the kali purum is especially applicable; and next follows a description of the cases of stricture in which the argenti nitras will be found useful. Some excellent practical remarks are made upon the treatment of those strictures in which a more considerable portion of the urethra is affected than usual.

The tenth chapter, "on the Removal of Strictures by Incision," merits particular attention. We know that some surgeons, of no mean celebrity, are either unacquainted with the operation of dividing the stricture through the perineum, or that they have formed an erroneous estimate of the great advantages of its occasional performance.

"Retention of Urine" is only spoken of as it occurs in consequence of stricture.

"The means by which we endeavour to relieve retention of urine, have for their immediate object the removal of local irritation, and production of general lassitude. If no success attend the accomplishment of these objects, an attempt is made to draw off the water by the introduction of the catheter: the last named measure seems, at first, so immediately calculated to effect our object, that it is too common to find surgeons, when called to a case of retention of urine, thinking of little else than the introduction of the catheter; and in instances where other means are thought of, this frequently precedes their employment. There is no doubt that occasionally the urine may, in this way, be at once evacuated; but to recommend it as a general practice appears to me very absurd, and argues a very superficial consideration of the causes on which the malady depends. The obstruction to the flow of urine is seldom so perfect, but that a few drops occasionally are allowed to pass; and if it be so, it does not result from the absolute closure* of the canal by the increase of the stricture, but from the irritation and spasm by

* "I had occasion once to puncture the bladder in a man aged seventy-three, whom I did not see until four days had elapsed without a drop of urine having passed from him. He was in the last stage of exhaustion. The bladder was felt extending even above the umbilicus, and no instrument could be passed to a greater distance than above three inches. The evacuation of a very large quantity of highly offensive and turbid urine was productive of temporary relief; but he gradually sunk, and in about twenty-four hours died. In addition to a much thickened bladder, enlargement of the third lobe, and an abscess in one lateral lobe of the prostate, I found a body, about the size of a large pea, close to the enlarged third lobe. I could by no means pass any instrument through the strictured portion. The canal seemed absolutely closed for about the third of an inch."

which it is affected. What, then, can be more irrational than the immediate attempt to introduce an instrument, which is never accomplished, even in health, without the production of more or less of these consequences? But it may be asked, do the results of practice accord with the suggestions of reason on this point? I should confidently answer in the affirmative. Every one knows that a practised hand may, in some cases, at once succeed in drawing off the urine; but, in the majority of these cases, I verily believe that, had the proper measures been employed, no instrument would have been necessary. As far as I have seen, the untimely attempt to introduce an instrument in severe cases of retention of urine, has not only been uniformly unsuccessful, but has very much increased the irritation on which the retention depends, and rendered the introduction of an instrument at the proper period infinitely more difficult, and in some cases impossible. I have seen many cases, where no instrument could be introduced, relieved by the judicious application of means presently to be mentioned. It is difficult to see any reason for the practice which I have endeavoured to reprobate, since there is no danger in waiting two or three hours, which will be sufficient for the institution of the proper practice. The bladder will contain a large quantity of urine:* it may ascend as high as the umbilicus, and yet no instrument be necessary for its evacuation. I should not have said so much on the early introduction of the catheter, had I not known that this injudicious practice is still pursued by men who are otherwise intelligent practitioners." (P. 136.)

To remove the local irritation, we must have recourse to leeches or cupping to the perineum, the warm bath, general blood-letting may be necessary, speedy evacuations from the bowels must be procured. Mr. M. is no great advocate for the use of opium in cases of retention of urine. Tartar emetic is considered a valuable medicine in all cases, "either where not a drop of urine can be expelled, or, as occasionally happens, where a little dribbling follows the administration of the other remedies, and the introduction of the catheter be still found impracticable."

"All the effects produced by the antimonium tartarizatum have an advantageous tendency. It produces lassitude; and, although absolute sickness is to be avoided, yet, should it occur, the prostration of strength will be more considerable. The profuse diaphoresis which it occasionally produces, is also desirable; since the secretion of urine usually goes on much less rapidly, and thus the bladder will not be so readily re-filled, should any urine be dribbling away, or, if otherwise, the quantity which it contains will not be so likely to receive any addition. The manner in which I have

* "Sabatier has recorded a case in which the bladder contained eighteen pints of urine.—See 'Médecine Opératoire.'"

usually given the antim. tartar. has been in warm water, in the proportion of about the half of a grain every twenty minutes or half-hour. The quantity, however, has varied considerably; that which I have mentioned is the average. I have sometimes given it with the calomel and jalap, but usually have allowed a little time to elapse between, for fear of producing sickness."* (P. 141.)

Should the foregoing plans prove unsuccessful, the surgeon should endeavour to introduce an instrument. With respect to the tobacco clyster, which Mr. Earle has extolled† in this complaint, the author's "present impression is, that the tobacco should be tried only where all other plans have failed, and where we have no other resource left than puncturing the bladder."

"Abscesses and Fistula in Perineo" are treated of in the eleventh chapter.

A few brief remarks are made on Stricture in the Female, and various interesting cases are detailed of irritable urethra, stricture, &c., which illustrate the particular circumstances which, in the opinion of the author, require the application of the different modes of treatment before mentioned.

"Stricture of the Rectum and Œsophagus" are next considered.

We particularly direct the attention of surgical students to the excellent comments offered by the author upon the important subject of the "Diagnosis of Tumors occurring at, or in the vicinity of, the Groin."

A short chapter on "Tracheotomy" concludes the work. Many cases which we have seen, both in public and private practice, convince us that surgeons in general, for what reason we have yet to learn, feel the greatest repugnance in performing this operation; even when the state of the patient leads to the conviction that any other treatment cannot succeed. Mr. Macilwain very correctly argues, that the numerous occasions on which the trachea has been opened where external tumors press on the tube, where foreign bodies have accidentally entered it, and lastly in asphyxia, afford incontestible evidence that the operation in itself is free from danger; at least, as much so as any other which surgeons are in the con-

* "Lest it should seem that I undervalue the power of opium in relieving irritation, I would here observe, that I do not recommend its use in ordinary cases, because there generally exist contrary indications, the fulfilment of which usually render the opium unnecessary, and with which the opium interferes. Where the bowels previously have been powerfully, but unsuccessfully, evacuated, a full dose of opium, that is to say, about fifty or sixty drops of laudanum, will, in some cases, be found successful in relieving the irritation, which produces the retention."

† Med.-Chir. Transactions, vol. vi.

stant habit of performing. His object here is only to speak of tracheotomy as forming part of the treatment of chronic laryngitis; and this is precisely the disease which we have more than once known terminate fatally, when the patient would at least have had a chance of recovery from this operation, and in which we have had to lament the unwillingness of the surgeon to perform it. Mr. Macilwain, in our opinion, very properly urges the necessity of the operation early in that disease, where other remedies have proved unsuccessful. We are sure that this is a part of surgical practice which still demands improvement, and, with the author, we are "convinced that, by a timely recourse to tracheotomy, many lives might have been saved which have been sacrificed to the fears of the patient, and sometimes, perhaps, to the indecision of the surgeon."

We must conclude our notice of the work with the following extract.

"I shall briefly give the results of my own experience as to the causes and symptoms of chronic laryngitis, the morbid appearances after death, the treatment which I would recommend previously, and the conditions which I consider necessary to the success of the operation. In most instances, the disease appeared to have been excited by some catarrhal affection, producing, at first, uneasiness in respiration, with the well-known vox rauca, which characterises the complaint. These symptoms have subsided, but have been easily again excited; and almost certainly, in a greater or less degree, on the approach of winter. As the warm weather returned, they have again retired; and in this way I have known some years elapse before the complaint has acquired the severity which induced the patient to seek professional assistance. When persons thus afflicted apply to a surgeon, there is seldom much time to be lost. The respiration has become habitually laborious, and accompanied with frequent paroxysms of irritation, which close the rima glottidis, and threaten immediate suffocation; and in one of these the patient expires. In some cases, the œsophagus sympathises with the larynx, which is evinced by occasional difficulty of deglutition. The larynx is sometimes tender on pressure. The morbid appearances consist of a much-thickened condition of the mucous membrane, which is in general more or less ulcerated, but not constantly. Sometimes there are considerable growths from it, or depositions exterior to it. These may be more or less firm in texture, or even afford the resistance given by cartilage. The effect resulting from either or all of these changes, is a considerable narrowing of the glottis: in some instances, the cartilages are perforated by ulceration, and certain ossified portions of them necrosed. I have also seen an ulcerated opening communicating with the pharynx; the whole surface of the larynx is usually lined by a layer of pus, with a light froth intermixed with it. Too often the lungs are found also diseased, having either puriform effusion into the ramifications of the bronchi, or suppurating tubercles in their parenchymatous structure. I have, however, observed other cases, where the lungs have been perfectly sound; nor do I believe that they generally become affected in consequence of disease of the larynx abstractedly, unless it has been allowed to go on for an improper period. The habitual impediment to respiration, consequent on chronic disease of the larynx, must certainly keep the lungs in a state of irritation and disturbance, well calculated to induce disease; and if, with such an exciting cause, there be any predisposition to pulmonary disease, some morbid condition of these viscera will most probably supervene in the laryngeal affection; but this, so far from militating against the performance of tracheotomy, is, in my opinion, (subject to the conditions to be mentioned,) a very strong argument for its early adoption. When the disease has proceeded so far as to produce ulceration, I fear that nothing but tracheotomy will save the patient; but where there has been tolerably conclusive evidence of change of structure, and where the symptoms have been characterised by considerable severity and suffering, I have succeeded, in a few instances, by a plan which has consisted chiefly of the employment of very vigorous local depletion, and the subsequent use of mercury. My experience induces me to attach quite as much importance to the former as to the latter part of the practice, since the mercury has too often failed when employed alone." (P. 320.)

There are few works we can so confidently recommend to the junior surgeon as the present. The author writes like a man of experience and judgment. He makes no effort to fill up space by the introduction of extraneous matter or useless speculation, but confines himself to a practical, and therefore useful, discussion of his subject.

---

*Practical Remarks on the Discrimination and Successful Treatment of Spasmodic Stricture in the Colon, considered as an occasional Cause of Habitual Confinement of the Bowels. Illustrated by Cases.* By JOHN HOWSHIP, Surgeon to St. George's Infirmary, Teacher of Surgery, &c.—8vo. pp. 62. Burgess and Hill, London, 1830.

THE author of this little treatise is well known to the profession as an observant and judicious practitioner. His present object is to show that the "large intestines are subject to a kind of permanent spasmodic contraction or stricture," and that such "stricture frequently gives rise to all the inconveniences induced by habitual and obstinate confinement of the bowels." Several of the cases brought forward by Mr. HOWSHIP, in his "Observations on the Diseases of the Lower Bowels," prove that the inferior parts of the intestinal tube are occasionally liable to be affected with spasm. Upon some occasions, Mr. H. has known a slight degree of irritation, arising from "a small abscess, situated at the verge of the anus, induce, in some parts of the bowel above, a degree of permanent spasm, of so determined a character, that, for the several months of its continuance, the patient, always previously used to a regular state of bowels, could never get them to act at all, without the assistance of medicine." One case is mentioned to illustrate this opinion, in which a "little hemorrhoidal abscess" was opened: the cavity, which was exposed, soon granulated and healed.

"But the remarkable and curious circumstance was, that from the day on which the operation was performed, his bowels thought proper again to transmit their contents with perfectly punctual regularity, unassisted by medicine; so that, to his great surprise, and without any change whatever, either in his diet or habits, he had now his one easy and regular motion every morning after breakfast, just as he had been accustomed to have a twelvemonth before." (P. 3.)

After some years' attention to the subject, Mr. H. is induced to believe that a general indisposition to action in the bowels, accompanied with a degree of occasional uneasiness or pain within some part of the abdomen, is, in many instances, brought on by a partial contraction in the intestine, "the effect of excessive muscular action, not the consequence of disease." The mere circumstance of an inactive condition of the bowels, thus induced by a little excess of muscular contraction in the affected part of the intestine, abstractedly considered, might be regarded as a matter of no material importance, in its bearings upon the general system.

"But the truth is, that one effect invariably induces another, until eventually a long chain of consequences present themselves, such as it would, at first, hardly have been suspected could possibly have risen out of a cause, in itself apparently so trifling.

"To advert to some of these consequences, and to convey an idea of their importance, it is only necessary to mention, in the first place, a state of stomach which absolutely prevents the patient from retaining or taking food or nourishment, solid or fluid; and, in the second, a constant sense of sorenesss or tenderness about the abdomen, so acute as entirely to prevent the patient from taking exercise, leaving the bed, or even turning in it; a state of irksome and continued suffering, protracted month after month, reducing life down to existence, and depriving the patient of every enjoyment or comfort that can render that existence, in any point of view, desirable.

"Independent of these more prominent characters, there are others, which, however, so far from assisting the practitioner, only serve still further to mask and conceal the true nature of the complaint. A degree of febrile excitement, white tongue, quick pulse, constant thirst, and occasionally a dry and heated skin; which, in addition to the foregoing symptoms, have repeatedly induced other persons, as well as myself, to believe that inflammation was going on, notwithstanding the means proper for relieving inflammatory action had been already adopted without the least beneficial effect." (P. 8.)

Mr. Howship is convinced, from his own observation, and the experience of others, that, when the intestinal canal is suffering under the effects of irritation, the large intestine, and especially the colon, is most commonly its particular seat. The following case fixed his attention to this subject, and confirmed his previous belief.

"May 21, 1826, Sunday, ten A.M. James Field, aged ten years, (8, George street, Grosvenor square,) was, eight days since, seized with violent pains in the bowels, and tenderness in the abdomen, for which Mr. D. of Oxford-street bled him from the arm, and the next day applied leeches to the abdomen; directing him, at the same time, proper medicines. The symptoms, much relieved, were indeed supposed to be nearly removed, but on the Wednesday they returned with violence, and so continued till Friday the 19th, when I was requested to see him. At this time the abdomen was so tender as not to bear the slightest examination, the weight of the bed-clothes or the least degree of motion.

"About every ten or fifteen minutes there was an increase of pain, which seemed to follow the course of the transverse arch and sigmoid flexure of the colon, with moaning and crying, and a pulse between 100 and 110. In the most painful part, towards the left side, there was, when lightly touched, the feeling of an indurated mass, apparently the size of half an orange, within the abdomen; this tumor being observed and felt by Mr. D. as well as myself. The abdominal parietes were relaxed, and moved freely over the surface of the tumor. Medicines were directed, but without any alleviation of the symptoms.

"On Saturday, a blister was directed to the abdomen, an aperient draught was also ordered, and a saline diaphoretic mixture, but without the least effect; the tongue covered with a thick fur; the skin hot and dry; the pulse quick and hard, though small; and the boy moaning, screaming, and crying, alternately, the day through.

"At nine P.M. we met, and finding the youth in no respect better, stated our opinion, that he was in considerable danger, and that in fact we had but little hope of his recovery. It was agreed, that at ten I should see him again, and try the effect of a copious warm injection, and that, if this gave no relief, he should be placed in the warm bath.

"At ten P.M. I found him decidedly worse, every symptom being aggravated in severity. The most careful introduction of the tube of the injecting apparatus produced a sudden increase of spasm, and a screaming fit. Even the slightest contact with the skin did the same thing. The fluid employed was warm groat gruel, about the temperature of 96°. I commenced the operation so slowly as to occupy five minutes in injecting the first half-pint. At first it was observable, that the slightest intrusion, even of the fluid, or the least degree of distention, aggravated the pain and spasm; but, as the volume of the contained fluid was increased, the bowel became evidently much more relaxed and less irritable, and the pains easier in the same proportion. Towards the conclusion of the experiment, when three pints were injected, relaxation prevailed to so decided a degree, that, while the youth said he was becoming very much easier, I perceived, to my surprise, that the previously dry and burning skin, now moist and glowing, was in a few minutes more covered with a copious perspiration; the pulse at the same time becoming fuller, softer, and in frequency subsiding down from 110 to seventy-five beats in the minute.

"He soon expressed an urgent desire to get up to the night-chair, where he passed the whole contents of the colon and rectum; which, as it was afterwards poured forth into another vessel, appeared to me a washing out of soft yellow feculent matter, which was most intolerably fœtid. A feculent matter, of so diffusive an odour as to render the apartment in a moment insupportably offensive, it was not difficult to perceive was quite adequate to produce, not only spasm and inflammation, but ulceration also, in the intestines.

"The next morning (Sunday, ten A.M.) we were told that he had fallen asleep just after I left him, and had continued to sleep, without medicine, the whole night, undisturbed. He also passed the whole of this day without the least return of pain, with a soft pulse at seventy-four, and much relief from the tenderness and tension of the abdomen. The feel of a tumor within one part of the abdomen could now no longer be perceived by the hand; the tongue was also becoming cleaner, and his only complaint was of hunger.

"I repeated the injection for several evenings; rather, however, in reference to the benefit derived from its first administration, than from any apparent tendency to a return of the symptoms. He was directed scarcely any medicine, but took nourishment freely. In three or four days he was up and well; and we took leave." (P. 12.)

From this case, Mr. H. perceived more clearly than he ever had before the great importance, in particular instances, of effectually relieving the lower bowels from the irritation of acrimonious contents. It enabled him also to perceive that, in this case at least, and probably in many others of a similar kind, the most painful, and perhaps the only spasm, was situated in the great intestine; and it showed also that, by the progressive introduction of a large volume of warm, emollient fluid, (provided the medical attendant will take the trouble to superintend its management himself, and watch its effect,) we have the power of inducing a gradual, but effectual and complete, relaxation in the contracted and painful portion of the intestine, although that portion may be beyond the reach of mechanical aid, administered by any other means.

"It may, perhaps, be said, that as the patient in this case recovered, I could not possibly know with certainty that the violent pains and spasms were situated in the colon, and there only. A moment's consideration, however, will be sufficient to demonstrate that the fact is otherwise. It will readily be granted that the introduction of the fluid, and washing out of the bowel, were the immediate cause of relief and recovery, as all the symptoms and sufferings directly ceased, and did not return. The patient appeared to be suddenly (that is, within a quarter of an hour) removed from a state of urgent danger to one of perfect safety and rapid convalescence. In the next place, we know that the valve of the colon would pretty well prevent the injection from passing beyond the great intestine; and we also know, that while, on the one hand, the valvular commencement of the colon is confined by its attachments to the right lumbar region, its opposite extremity, the sigmoid flexure, upon the other, is also bound down to the left loin; and that the only part below is the rectum. It follows therefore inevitably, that the colon, being the only bowel so situated as to pass invariably across the abdomen, was the only part of the intestinal canal that could have been the seat of the entire and complete relief by the means employed, and, consequently, it must also have been the seat, and only seat, of the complaint." (P. 18.)

Mr. Howship next proceeds to relate cases, which he believes afford proof that the large intestines are occasionally subject to a permanent kind of spasm: "so far, at least, permanent, as to continue for many years; yet so far spasmodic, as to admit, under proper treatment, of being entirely relieved and relaxed in a few weeks." From the detail of these cases, the symptoms which generally attend upon a permanent spasmodic contraction in the great intestine, may be well understood.

"In the first place may be mentioned, the constant or habitual tendency to confinement in the bowels, in consequence of which they require a greater degree of excitement, to render them active, than is wanted in a state of perfect health. This inconvenience is such, that, at times, the most active purgative is scarcely sufficient to ensure relief. This symptom may exist for several, or many years, before any other cause of complaint appears to arise.

"Sooner or later, however, occasional local feelings, first of uneasiness, then of pain, perhaps of a decidedly acute description, are induced; in most instances confined to one and the same part, attended with a sense of soreness when the seat of the complaint is examined or pressed upon, or when the chest is moved upon the pelvis.

"In the progressive course of this disorder, the stomach is in every case more or less deranged; and in some instances, extreme irritability of this organ takes the lead of every other symptom, and becomes alarming, from the total suspension of its functions, threatening to destroy life by inanition.

"The diffused sense of soreness and tenderness of the abdomen, with increased distress from the extent or degree of local pain, is liable to bring on attacks of constitutional disturbance and irritative fever; modified in its symptoms by the peculiarities of its cause. The thirst is constant and considerable, and the tongue (as I have seen it) white; although any prevailing bilious irritation will of course influence and alter this appearance. The pulse is quick and small, and, unless there is decided heat of skin, it will be soft also. Where the local pain becomes very acute, the reaction of the system will be violent in proportion, the pulse becoming hard, the skin hot and dry, and the thirst extreme.

"A great diversity in the occasional combination and course of the symptoms in these cases, will, I believe, depend on the accidental coincidence of irritation with spasm. It may be said here, and it is perfectly true, that irritation must exist, in order to spasm being induced; and it may perhaps be therefore concluded, that, the effect being acknowledged to be present, the cause must be supposed to be so too. If, however, it be correct to admit this conclusion, it must, I conceive, be allowed only to a very limited extent.

"The irritation, for example, may bear no relation at all, in permanence or severity, to the spasm; the former being so slight in degree, and so independent of pain, as for many years to have passed entirely unheeded by the patient; while the latter, constantly and obstinately present, shall totally suspend all passage of contents through the alimentary canal, unless when excited by the action of drastic purgatives.

"Again, on the other hand, the spasm may bear no comparison with the irritation, the occurrence even of transitory spasmodic constriction having never prevented the regular and punctual transmission of the contents of the bowels, in many cases in which the effects of irritation had induced all their worst consequences: chronic inflammation, extensive ulceration, sometimes epileptic convulsions, and eventually destruction of life.

"The diagnosis of this peculiar affection may, upon the first glance, appear involved in much obscurity: a little care, however, will, I believe, enable the attentive practitioner to discriminate this complaint with satisfactory and perfect precision.

"The long-continued sense of soreness, and perhaps pain or uneasiness, most commonly confined to some one part of the abdomen, the established habit of confinement in the bowels, the occasional increase or diminution in the acuteness of the pain and tenderness, or the accession of habitual irritability of stomach and constitutional disturbance, are symptoms that I should think, in most instances, would be sufficient to identify the nature of the complaint; although I consider that it is the invariable and obvious influence produced upon the symptoms, under the operation of distending the bowel, which alone affords that direct evidence, on which implicit reliance may be placed, in determining the existence and particular habits of this peculiar derangement." (P. 39.)

The necessity of greater attention in the administration of enemas, is very properly insisted upon. The safety of patients not unfrequently depends upon these remedies; and yet, in the majority of cases, their management is confided to an ignorant and awkward nurse.

In concluding his remarks on distention of the great intestine, for the removal of spasmodic contraction of the gut, Mr. Howship observes, that, in order to prove successful, the operation requires,

" 1st. That the fluid should be introduced with the least possible disturbance to the sphincter, and consequently that the tube conveying it should be always passed carefully.

" 2d. That the fluid used should be of one and the same degree of warmth upon every occasion, the temperature to be preferred being that of blood-heat, or 96° of Fahrenheit.

" 3d. That the fluid be introduced at first very slowly, and the instrument used so easy and free in its action, that, as the fluid flows into the bowel, the hand of the operator may be sensible of any increasing resistance to further expansion of the gut, independent of any thing the patient may say as to his own feelings.

" 4th. The quantity introduced should be accurately observed as the operation proceeds, in order that, as the fluid cannot make its way beyond the valve of the colon, the maximum in volume may never exceed that which may reasonably be supposed equal to the healthy capacity of the intestine; and when introduced, it should be there retained for a certain time, to produce the desired effect; for which purpose I should say an interval of from fifteen to thirty minutes may be sufficient.

" 5th. That, as far as my present limited experience on the subject enables me to judge, it appears that, in order to its ultimate success, the operation should be repeated daily for a period of three weeks, or perhaps a month." (P. 48.)

